# Co‐designing an intervention to improve the process of deprescribing for older people living with frailty in the United Kingdom

**DOI:** 10.1111/hex.13669

**Published:** 2022-11-24

**Authors:** Jonathan Silcock, Iuri Marques, Janice Olaniyan, David K. Raynor, Helen Baxter, Nicky Gray, Syed T. R. Zaidi, George Peat, Beth Fylan, Liz Breen, Jonathan Benn, David P. Alldred

**Affiliations:** ^1^ School of Pharmacy and Medical Sciences, Faculty of Life Sciences University of Bradford Bradford UK; ^2^ NIHR Yorkshire and the Humber Patient Safety Translational Research Centre, Bradford Institute for Health Research Bradford UK; ^3^ School of Healthcare University of Leeds Leeds UK; ^4^ Alliance Manchester Business School, Faculty of Humanities University of Manchester Manchester UK; ^5^ Department of Pharmacy, School of Applied Sciences University of Huddersfield Huddersfield UK; ^6^ HPS Pharmacies EBOS Group Docklands Victoria Australia; ^7^ Department of Health Sciences University of York York UK; ^8^ School of Psychology University of Leeds Leeds UK

**Keywords:** aged, deprescribing, frailty, polypharmacy, primary health care, referral and consultation

## Abstract

**Background:**

In older people living with frailty, polypharmacy can lead to preventable harm like adverse drug reactions and hospitalization. Deprescribing is a strategy to reduce problematic polypharmacy. All stakeholders should be actively involved in developing a person‐centred deprescribing process that involves shared decision‐making.

**Objective:**

To co‐design an intervention, supported by a logic model, to increase the engagement of older people living with frailty in the process of deprescribing.

**Design:**

Experience‐based co‐design is an approach to service improvement, which uses service users and providers to identify problems and design solutions. This was used to create a person‐centred intervention with the potential to improve the quality and outcomes of the deprescribing process. A ‘trigger film’ showing older people talking about their healthcare experiences was created and facilitated discussions about current problems in the deprescribing process. Problems were then prioritized and appropriate solutions were developed. The review located the solutions in the context of current processes and procedures. An ideal care pathway and a complex intervention to deliver better care were developed.

**Setting and Participants:**

Older people living with frailty, their informal carers and professionals living and/or working in West Yorkshire, England, UK. Deprescribing was considered in the context of primary care.

**Results:**

The current deprescribing process differed from an ideal pathway. A complex intervention containing seven elements was required to move towards the ideal pathway. Three of these elements were prototyped and four still need development. The complex intervention responded to priorities about (a) clarity for older people about what was happening at all stages in the deprescribing process and (b) the quality of one‐to‐one consultations.

**Conclusions:**

Priorities for improving the current deprescribing process were successfully identified. Solutions were developed and structured as a complex intervention. Further work is underway to (a) complete the prototyping of the intervention and (b) conduct feasibility testing.

**Patient or Public Contribution:**

Older people living with frailty (and their informal carers) have made a central contribution, as collaborators, to ensure that a complex intervention has the greatest possible potential to enhance the experience of deprescribing medicines.

## 1 INTRODUCTION

Older people living with frailty are vulnerable to harm because of age‐related breakdown in physiological systems and the failure of homoeostasis.^1^ Specifically, nonfrail older people have greater tolerance to adverse drug effects (ADEs),^2^ and frailty is a better predictor of medicines‐related harm than chronological age.^3^ Therefore, targeting frail older people has become an important focus internationally for medicines optimization and deprescribing interventions to reduce polypharmacy.^2^

In this context, polypharmacy is the concurrent use of multiple medicines, usually defined as the use of five or more medicines daily.^4^, ^5^ The King's Fund refers to ‘appropriate’ and ‘problematic’ polypharmacy to differentiate between safe and potentially harmful combinations of medicines.^6^ Polypharmacy is problematic when the potential risks of use outweigh the expected benefits. In older people living with frailty, this can lead to higher healthcare costs and preventable harm such as adverse drug reactions,^7^, ^8^, ^9^, ^10^, ^11^ hospitalizations,^12^, ^13^, ^14^ falls,^15^, ^16^, ^17^ lower levels of adherence^6^, ^18^ and mortality.^19^

The World Health Organization (WHO) has a Medication Without Harm initiative, with targets to reduce harm from problematic polypharmacy.^20^ In the United Kingdom, the new general practice (family doctor) contract is tackling polypharmacy by ensuring that periodic Structured Medication Reviews (SMRs) are conducted in line with the National Health Service (NHS) Long‐term Plan and there is a clear process for deprescribing.^21^

Deprescribing is defined as: ‘the systematic process of identifying and discontinuing medicines in instances in which existing potential harms outweigh existing or potential benefits within the context of an individual patient's care goals, the current level of functioning, life expectancy, values, and preferences’.^22^ It is increasingly recognized as a strategy to reduce problematic polypharmacy.^23^, ^24^ There are, however, professional and service user reported barriers to deprescribing.^10^, ^25^ Stopping prescribed medicines is often complex so it requires shared decision‐making and mutual understanding.

Guidelines for shared decision‐making recommend actions before, during and after the clinical consultation to ensure full service‐user engagement in their care. These actions may be enhanced by: the involvement of a supporting person (e.g., family member) and links to reliable health information.^26^ A person‐centred approach to deprescribing ensures successful therapeutic change.^5^ There are reasons to believe that greater sensitivity to lived experience can enhance service delivery for people living with frailty.^27^ There are also moral reasons to engage vulnerable people in the design of public services,^28^ which include an assumption of capacity to make informed decisions and a desire for the experience of service delivery to be positive. In a health context, people need information about the potential risks and benefits of treatment options at a level of detail that helps them to make an informed choice with professional guidance.^29^

One person‐centred approach to improving healthcare services is experience‐based co‐design (EBCD).^30^, ^31^, ^32^, ^33^, ^34^, ^35^, ^36^, ^37^, ^38^ EBCD is a narrative‐based participatory method, which brings together professionals and service users to collaboratively co‐design local services. This can be used instead of (or alongside) more traditional approaches to service improvement (e.g., Plan Do Study Act cycles) with a particular focus on the user experience of service delivery.^39^

Traditionally, EBCD is conducted in one organization that initiates and implements the process, and then solutions are implemented locally. There is variation in the use of EBCD,^40^ and co‐design alone does not necessarily solve all healthcare delivery problems.^41^ We have previously designed an intervention using researcher‐driven EBCD and implemented this in a clinical trial to improve medicines management during discharge for heart failure patients.^30^, ^42^ The work described in this paper had similar intentions in a different context.

In preparation, we had already explored processes for, and the lived experience of, deprescribing in interviews with older people, their informal carers and professionals. Drawing on this work, we identified six themes related to barriers and facilitators of deprescribing.^43^ This is analogous to the first phase of EBCD. In response to these themes, in this current phase of work, we completed a modified EBCD process to develop an intervention to address problematic polypharmacy in primary care. This approach builds on the strengths of traditional EBCD to develop a new intervention based on an agreed model of service delivery.

The value of careful engagement with vulnerable people to improve services has been recognized,^44^ but there have been no published studies drawing on the experiences of users and professionals to collaboratively design and evaluate a process for deprescribing in primary care settings. This study aimed to co‐design an intervention to improve deprescribing processes for older people living with frailty who were receiving primary care in the English NHS.

## 2 METHODS

**Figure 1:**
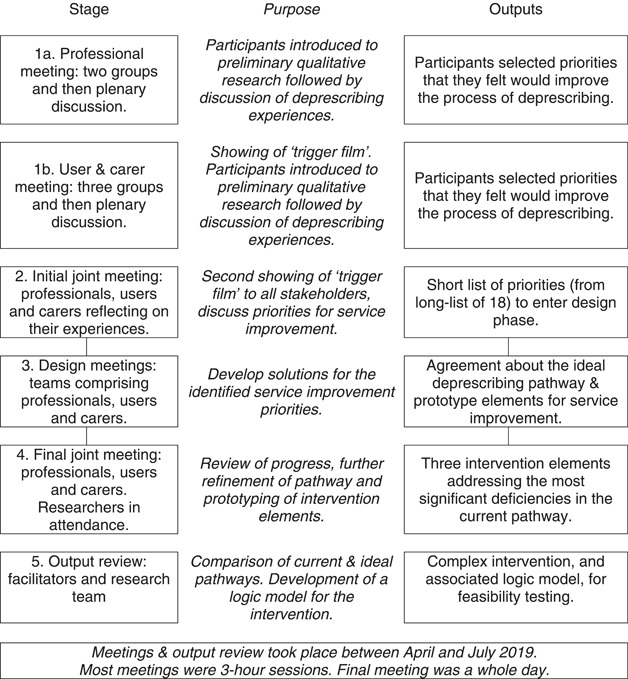
Intervention development meetings: purpose and intended outputs

Usually, EBCD includes thematic analysis of interviews with stakeholders; preparation of a trigger film to use in stakeholders' meetings; collaborative problem identification and priority setting and the co‐design of solutions (Figure 1). Elsewhere, we have described our qualitative research that forms the first phase of EBCD in this case, that is, gathering information from service users and professionals about deprescribing experiences.^43^

In this phase, we completed the EBCD process and also integrated it with Medical Research Council (MRC) guidelines for the development of complex research interventions.^45^ This modification of EBCD is in line with our previously described process for the design of complex interventions.^30^, ^42^ The link between the phases of work is a trigger film summarizing the emotional touchpoints in the process of deprescribing from an older person's perspective. This film is used to focus discussions in the stakeholder meetings described below.

The objectives of this phase of work were to:

(1) hold a series of stakeholder meetings for service users and professionals to further discuss problems with deprescribing and identify priorities for service improvement;
(2) run design meetings to work from the agreed priorities and towards solutions (changes in process) that will enhance the experience of deprescribing with the intention to improve safety and effectiveness;
(3) build these solutions into a complex intervention that can be tested in further phases of work;
(4) produce a logic model that will support the future implementation and evaluation of the complex intervention.

### 2.1 Planning for stakeholder meetings

The inclusion criteria for stakeholders were:

(1) people 65 years and older identified as living with frailty or at risk of frailty (a recorded diagnosis and/or electronic Frailty Index [eFI] > 0.12) who were experienced in the daily management of multiple medicines for co‐morbidities^43^, ^46^;
(2) family members, or others, supporting these people on a daily basis in an unpaid or voluntary capacity;
(3) primary care healthcare professionals—pharmacists, general practitioners and nurses, with experience in deprescribing for older people.

In the United Kingdom, primary care pharmacists now work as part of a multidisciplinary team in medical general practice. We did not engage (in this study) with community (retail) pharmacists. To supplement the core research team, an experienced EBCD trainer (H. B.) and a group‐work facilitator (N. G.) were commissioned. An EBCD training event was held for the whole team with the trainer (H. B.) who was accredited by the Point of Care Foundation. Users, carers and professionals were approached using a number of channels: Yorkshire and Humber Academic Health Science Network (AHSN); West Yorkshire NHS Research & Development office; Yorkshire and Humber Patient Safety Translational Research Centre; citizens' groups in Bradford, for example, Age UK; Community Pharmacy West Yorkshire (CPWY); the service user group in the University of Bradford's Faculty of Health Studies, and participants from the qualitative interview stage, for example, professionals from two local medical general practices.

Letters inviting people to separate initial meetings were circulated to users and informal carers; and professionals. Before the meetings, any questions were answered by telephone. At the initial meetings, the whole design process was described in detail and agreements to continue with participation were confirmed. Participants were free to attend as many sessions as they were able. The initial professional meeting was held on 29th April 2019 and the final prototyping with stakeholders was held on 3rd June 2019. All other meetings were held in this window.

### 2.2 Priority setting meetings

At the initial professional meeting, two working groups shared information about their workload and clinical case studies. At the initial user and carer meeting, discussions were facilitated in three working groups. The trigger film showing older people talking about their experiences was viewed then experiences of deprescribing were shared and discussed. Each of these initial (segregated) stakeholder meetings produced a long list of priorities for service improvement.

At a joint meeting for all stakeholders, the trigger film was reshown and the long lists of priorities previously agreed upon were shared. People were set the task of jointly agreeing, by facilitated discussion and debate, a short list of priorities.

### 2.3 Intervention design meetings

From this point onwards the users, informal carers and professionals met as a design team. Volunteers to join the design team were sought and confirmed by the group at the end of the joint meeting. At the first design team meeting, facilitators acted as scribes for two working groups. Thoughts and ideas were captured on sticky notes so that they could be sorted and arranged. At the second design team meeting, notes from the first meeting were presented for validation and any disagreements were resolved by discussion. Existing elements of good practice were noted and the participants worked towards interventions that would improve care.

In the original plan, a final meeting of users, carers and professionals was intended as a celebration and a space to present intervention prototypes. However, progress made to that point was presented to the whole group (those who had also attended the second design meeting) and then prototyping of interventions continued in three subgroups with facilitators. Various creative resources were made available including coloured pens, paper/card, plastic building blocks, blank speech bubbles, sticky notes, flip charts and wall space. Printed summaries of prior outputs were available for reference.

Since this work was a development activity (leading to future research) the people involved were considered to be collaborators or co‐investigators and therefore, ethical approval was not sought. Personal information about people was not captured. Discussions were not recorded or subjected to thematic or content analysis. Prior ethical approval had covered activities up to and including the creation and viewing of the trigger film.

## 3 RESULTS

The number and type of people contributing are summarized in Table 1. Table 1 aligns with Figure 1, which shows how the outputs of each EBCD stage fed into the next stage. Note that EBCD is iterative and incorporates ongoing participant validation. The ending of one stage is the beginning of the next. In this way, a consensus is built, and peoples' ideas are taken forward with fidelity. However, the initial professional and user meetings are not linked.

**Table 1:** Number and type of participants at each stage

Stage of process	Participants
Professional meeting	1 medical general practitioner, 1 hospital pharmacist, 7 primary care pharmacists. 2 facilitators.
User and carer meeting	5 informal carers and 9 older people. 3 facilitators.
Initial joint meeting	3 primary care pharmacists, 3 informal carers and 5 older people. 3 facilitators.
Design meetings	3 primary care pharmacists and 9 older people or informal carers. 2 facilitators.
Final joint meeting	3 facilitators. Participants from the Design Meetings.
Output review	Meeting facilitators and members of the academic team.

### 3.1 Priority setting meetings

In the initial professional meeting, the importance of good relationships with older people was agreed and some high‐risk medicines were identified. Professionals also noted risks associated with different parts of the care pathway and how significant safety incidents were managed. The risks associated with making and monitoring deprescribing decisions were noted, as was the need for appropriate record keeping.

It was clear that the professionals had a shared experience of problems when deprescribing and were keen to help colleagues in similar situations. Professionals noted that older people could be resistant to deprescribing if they trusted the initial prescriber and that therapeutic alternatives were sometimes lacking. Exacerbations or new diagnoses could provide opportunities for a medication review. Professionals agreed on a long list of 12 priorities which they felt would improve the process of deprescribing:

1. Team‐based approach and clarity of roles and responsibilities.
2. Information available to healthcare professionals.
3. Information available to older people and informal carers.
4. Service user engagement and empowerment.
5. Implementation of triggers in the system to identify opportunities to optimize medicines, of which deprescribing is one component, for example, admission to hospital or discharge.
6. Communication at transitions of care (with other professionals and with older people): handovers.
7. Follow‐up after medicines are stopped.
8. Standardization of guidelines for deprescribing.
9. Clear plan for each medicine prescribed when they are prescribed: agreeing on goals.
10. Skills of healthcare professionals: opportunities to reflect and learn about the process, what went well and what could be improved.
11. Skills of older people: opportunities to reflect on the process, what went well and what could be improved.
12. Time required to stop medicines.

In the initial user and carer meeting, difficulties in working relationships between hospital consultants, GPs and community pharmacists were noted. Older people were sometimes unsure about who to ask questions to or where to seek clarification about plans for care. Older people were aware that medicines had both risks and benefits.

Older people and carers agreed to a long list of six priorities:

1. Two‐way discussions incorporating personal views and priorities.
2. Following‐up and monitoring of changes should be organized.
3. User‐professional relationships and familiarity with professionals should be improved: ‘no decision about us without us’.
4. Advance information should be provided about medicines and proposed changes.
5. Alternatives to medicines should be considered.
6. User access to peer support should be noted and carer views on change considered.

At the joint meeting, the trigger film had a powerful effect on professionals who were viewing it for the first time and in the presence of older people. The highlighted themes, spoken about by older people in the film, included clarity of technical information, transparency of processes and the need for trust in consultations. Older people and carers at the meeting expressed the need for some flexibility around decision‐making to account for uncertainties, domestic circumstances and the variability of health status. Through discussion at the joint meeting, the overall long list of 18 priorities was reduced to a short list of two priorities, which the design groups then addressed. These were:

- Two‐way conversations/discussions: attitudes, prior knowledge, preparation, skills, expectations—described as ‘general culture changes needed to form the context to a deprescribing decision’.
- The process of stopping medicines: the steps to take before, during and after consultation including follow‐up.

### 3.2 Intervention design meetings

**Figure 2:**
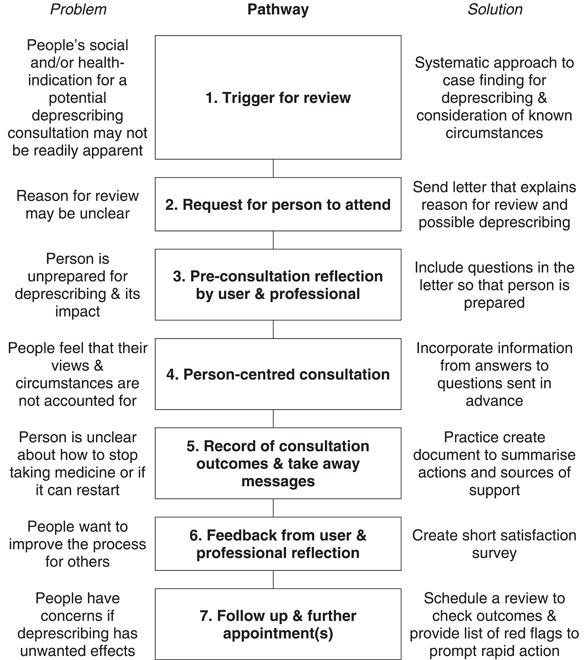
Simplification of the ideal deprescribing pathway with perceived problems and proposed solutions (an output from the design meetings)

In the design meetings, the initial discussion was free ranging around preparation for deprescribing, the actual consultation and follow‐up. Two planned working groups were quickly merged and their outputs linked because they were converging. An ideal deprescribing process was outlined (simplified in Figure 2). The resources available for prototyping interventions were limited, so the co‐design groups tried to made progress where they thought the capacity for benefit was greatest.

More time for prototyping was arranged by repurposing the proposed celebration meeting to some extent. People developed prototypes for three interventions that were suggested enhancements to the deprescribing process:

(1) An invitation letter to a deprescribing consultation providing information about the purpose of the consultation and encouraging older people to prepare questions for professionals.
(2) A ‘take‐away’ for the end of the consultation listing: the agreed next steps, monitoring to ensure the safety of deprescribing, and how the effectiveness of any decisions made would be reviewed.
(3) A satisfaction survey for older people to complete and provide feedback for professionals about the consultation process.

### 3.3 Review of intervention design outputs

In subsequent researcher meetings, differences between the current deprescribing pathway and the ideal pathway (Figure 2) were further explored and discussed. Seven major differences were noted between the current deprescribing pathway and the ideal pathway that had been generated. Since prototyped interventions addressing three of these differences had already been developed, this left a further four interventions that still required initial prototyping:

(1) Professional training: focussed on consultation skills and shared decision‐making.
(2) Ensuring the consultation has a clear agenda—the older person's initial feelings and any concerns about action points should both be addressed.
(3) Signposting information for further postconsultation support and updating any user‐held records.
(4) Giving the older person a list of triggers (red flags) that would require rapid follow‐up and monitoring repeat prescription requests or missed appointments.

**Figure 3:**
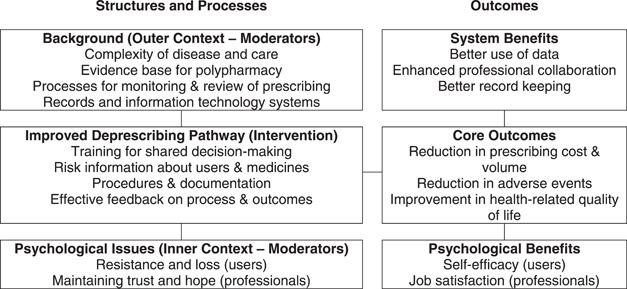
Simplified logic model for the proposed intervention. Connecting lines suggest influence or interaction. However, the model is dynamic rather than static and influence is not unidirectional.

We refined the existing prototypes and generated an overall logic model^47^ for a complex intervention (combining the seven simple interventions) structured around the ideal pathway (Figure 3). Usually, EBCD outputs would be implemented locally and refined by iterative cycles of service improvement. We recognized that elements of the ideal pathway were present in current NHS practice even if not fully expressed. Pathway improvements could also be implemented in different ways, for example, written materials may be physical or electronic. Therefore, we proposed that prototyping continued (after this co‐design process) with partners in primary care to develop tools and resources that could be implemented flexibly to meet local needs and create the ideal pathway.

## 4 DISCUSSION

The final priorities (most important problems) identified by our collaborators were the clarity of all stages in the deprescribing processes and the quality of one‐to‐one consultations. Three simple interventions were prototyped to address these priorities and four more simple interventions were identified. Working from these simple interventions, a complex healthcare intervention (Figure 2) and a supporting logic model (Figure 3) were created. Further work is ongoing to conduct feasibility testing with primary care partners before full evaluation.

Our work has been conducted in close partnership with older people living with (or at risk of) frailty and their informal carers. In the NHS, the eFI is used as a risk stratification tool to identify if people are likely to be fit or living with mild, moderate or severe frailty.^47^ This risk stratification allows body system reviews and medication reviews to be targeted for people who are at the highest risk of adverse events and, therefore, most likely to benefit from deprescribing.

If primary care professionals are working efficiently to improve the safety of medicines used for those identified as living with (or at risk of) frailty, then the incidence of deprescribing events should appropriately increase. The intention of deprescribing is to reduce problematic polypharmacy, however, this co‐design work has shown that the process of deprescribing is itself problematic: requiring further optimization and evaluation. Any changes to medicines that have been prescribed for a long period can carry risks as well as benefits, such as adverse drug withdrawal events.^48^

Older people and carers understand the need for deprescribing in general, but the six long‐listed user priorities (from the initial user and carer meeting) in this study focused on the themes of information and relationships. Older people and carers want to be told the rationale for medicine changes and have the opportunity to express how these changes will influence their daily lives. People living with frailty also want reliable access to peer and professional support. These user priorities (from co‐design) reflect the facilitators of deprescribing identified in preparatory work.^43^

The professional priorities identified often supported, and do not fundamentally conflict with, the user priorities. However, professionals also identified the importance of: skill mix; clear roles and responsibilities; triggers to action; planning and guidelines. Professionals seek constructive engagement with users and carers. However, the working environment is already complex and there are competing demands on professional time. The proposed ideal pathway (Figure 2) seeks to structure a process around some elements that already exist in practice but may not be consistently delivered. These professional priorities (identified in stakeholder meetings) reflect more of the barriers to deprescribing identified in preparatory work.^43^

Structured and routine engagement with users, which privileges the lived experience, brings them into the healthcare system as self‐managers and monitors. Those living with frailty are (by definition) vulnerable, however, they are not helpless or hopeless. Here they have made an important contribution, as collaborators and co‐designers, to ensure that a complex intervention has the greatest possible potential to enhance the experience of healthcare delivery.

In the ideal pathway, a flow of information is created, integrated and managed. There are stages of information gathering, clinical decision‐making and information giving; akin to consultation skills guides such as the Calgary‐Cambridge model^49^ and the derived Medicines Related Consultation Framework (MRCF).^50^ However, the flow of information includes checks, balances and feedback loops, meaning that the deprescribing process could be paused or reviewed. One key characteristic of this pathway is that at each stage, older people are actively engaged, shared decisions are made and intended outcomes are clarified. The pathway also recognizes that, for users, medicines taking and deprescribing take place in a psychological and social context. A process that potentially addresses concerns and integrates social support is more likely to be effective and enhance user satisfaction. Co‐design has allowed us to build on the current (implicit) deprescribing process that preparatory work had previously mapped out.^43^

The ideal pathway now shown (Figure 2) is transparent, fully defined and designed to enhance collaboration at each step. In England, SMRs will be undertaken by pharmacists working in Primary Care Networks (PCNs) and our intervention will be feasibility tested within this context.^51^ A study has shown that deprescribing for older people with type 2 diabetes is feasible, safe and may improve quality of life.^52^ The risks and benefits of deprescribing will vary condition by condition and we have only developed a generic pathway. When people lack the capacity to engage, then their carers' views may also be considered.^53^

A more diverse mix of healthcare professionals could have strengthened our study; however, as primary care pharmacists will take the lead role in SMRs the relatively high number of pharmacists who were part of the EBCD process has strengthened our process model for adoption. It was recognized that a relatively small group of participants may miss opportunities for innovation and creativity, especially in a short timescale. However, the overarching aim of EBCD is not necessarily one of generalizability. Rather, our findings will support the feasibility testing of flexible tools and processes to enhance existing consultation processes in primary care.

## 5 CONCLUSION

Previous work demonstrates that deprescribing of potentially inappropriate medicines in older people living with frailty has the potential to prevent ADEs and improve peoples' quality of life worldwide. However, we show that deprescribing itself must be carefully managed to optimize effectiveness and minimize risks. Our pathway outlines a person‐centred, clinician‐facilitated approach to deprescribing consultations in primary care, which is also supported by a recent systematic review.^54^ Our study further demonstrates that EBCD can work across multiple general practices as part of a programme of research to develop a person‐centred deprescribing process. This has the potential to improve service efficiency and user outcomes. In keeping with the unique characteristics of EBCD, users, informal carers and professionals were best placed to identify areas for improvement in the current pathway for medication reviews and deprescribing. Collaborative intervention design ensures that changes address the needs and concerns of all stakeholders.

## AUTHOR CONTRIBUTIONS

Jonathan Silcock and David K. Raynor led and directed the project. Iuri Marques and Janice Olaniyan managed and delivered the project. Helen Baxter and Nicky Gray were EBCD facilitators and supported project delivery. Syed T. R. Zaidi supported project delivery, was an EBCD facilitator and also participated in intervention review. George Peat, Beth Fylan, Liz Breen and Jonathan Benn led and managed supporting projects, contributed to the review and refinement of the intervention and edited the manuscript. David P. Alldred leads the programme of work that this project is part of, contributed to the review and refinement of the intervention, and edited the manuscript.

## CONFLICT OF INTEREST

The authors declare no conflict of interest.

## Data Availability

The data that support the findings of this study are available from the corresponding author upon reasonable request.
